# Host-Viral Interactions at the Maternal-Fetal Interface. What We Know and What We Need to Know

**DOI:** 10.3389/fviro.2022.833106

**Published:** 2022-03-24

**Authors:** James H. Girsch, Maria C. Mejia Plazas, Amanda Olivier, Mohamed Farah, Dawn Littlefield, Supriya Behl, Sohan Punia, Reona Sakemura, Jack R. Hemsath, Andrew Norgan, Elizabeth A. L. Enninga, Erica L. Johnson, Rana Chakraborty

**Affiliations:** 1 Department of Pediatric and Adolescent Medicine, Mayo Clinic College of Medicine, Rochester, MN, United States,; 2 Mayo Clinic Graduate School of Biomedical Science, Rochester, MN, United States,; 3 Department of Microbiology, Biochemistry, and Immunology, Morehouse School of Medicine, Atlanta, GA, United States,; 4 Department of Pediatric Research, Mayo Clinic, Rochester, MN, United States,; 5 Department of Hematology Research, Mayo Clinic, Rochester, MN, United States,; 6 Department of Infectious Diseases Research, Mayo Clinic, Rochester, MN, United States,; 7 Department of Laboratory Medicine and Pathology, Mayo Clinic College of Medicine, Rochester, MN, United States,; 8 Department of Immunology, Mayo Clinic College of Medicine, Rochester, MN, United States,; 9 Department of Obstetrics and Gynecology, Mayo Clinic College of Medicine, Rochester, MN, United States

**Keywords:** placenta, HIV-1, human cytomegalovirus, Zika, vertical transmission, cytotrophoblast, Hofbauer cell, natural killer cell

## Abstract

In humans, the hemochorial placenta is a unique temporary organ that forms during pregnancy to support fetal development, gaseous exchange, delivery of nutrition, removal of waste products, and provides immune protection, while maintaining tolerance to the HLA-haploidentical fetus. In this review, we characterize decidual and placental immunity during maternal viral (co)-infection with HIV-1, human cytomegalovirus (HCMV), and Zika virus. We discuss placental immunology, clinical presentation, and epidemiology, before characterizing host susceptibility and cellular tropism, and how the three viruses gain access into specific placental target cells. We describe current knowledge on host-viral interactions with decidual and stromal human placental macrophages or Hofbauer cells, trophoblasts including extra villous trophoblasts, T cells, and decidual natural killer (dNK) cells. These clinically significant viral infections elicit both innate and adaptive immune responses to control replication. However, the three viruses either during mono- or co-infection (HIV-1 and HCMV) escape detection to initiate placental inflammation associated with viral transmission to the developing fetus. Aside from congenital or perinatal infection, other adverse pregnancy outcomes include preterm labor and spontaneous abortion. In addition, maternal HIV-1 and HCMV co-infection are associated with impaired fetal and infant immunity in postnatal life and poor clinical outcomes during childhood in exposed infants, even in the absence of vertical transmission of HIV-1. Given the rapidly expanding numbers of HIV-1-exposed uninfected infants and children globally, further research is urgently needed on neonatal immune programming during maternal mono-and co-infection. This review therefore includes sections on current knowledge gaps that may prompt future research directions. These gaps reflect an emerging but poorly characterized field. Their significance and potential investigation is underscored by the fact that although viral infections result in adverse consequences in both mother and developing fetus/newborn, antiviral and immunomodulatory therapies can improve clinical outcomes in the dyad.

## INTRODUCTION

Healthy human placentation is vital for fetal viability and development in pregnancy. In order to optimize this process, hemochorial placental changes occur during gestation, which address a myriad of functions specific to survival of the fetus. Ongoing adaptations include gaseous and nutrient exchange, removal of waste products, and immune protection while maintaining tolerance to the HLA-haploidentical fetus (1, 2). The maternal-fetal interface (MFI), which is made up of the maternal decidua and fetally-derived placenta offsets perinatal transmission of invasive pathogens throughout gestation…most of the time. The specific mechanisms by which immune homeostasis is maintained, paradoxically enabling survival of genetically distinct offspring, while preventing invasive infection of the fetus is only beginning to be understood. An extensive discussion of immune tolerance during pregnancy is beyond the scope of this review, which focuses on how innate and adaptive responses play a key role in restricting maternal viral infections at the MFI from entry into the fetal circulation. This review discusses how three key viruses (Zika virus—ZIKV, human immunodeficiency virus-1—HIV-1, and human cytomegalovirus—HCMV) either during mono- or co-infection (HIV-1 and HCMV) escape detection by adaptive and innate immune responses at the decidua and placenta to initiate and promote inflammation associated with viral transmission to the developing fetus.

**FIGURE 1 |:**
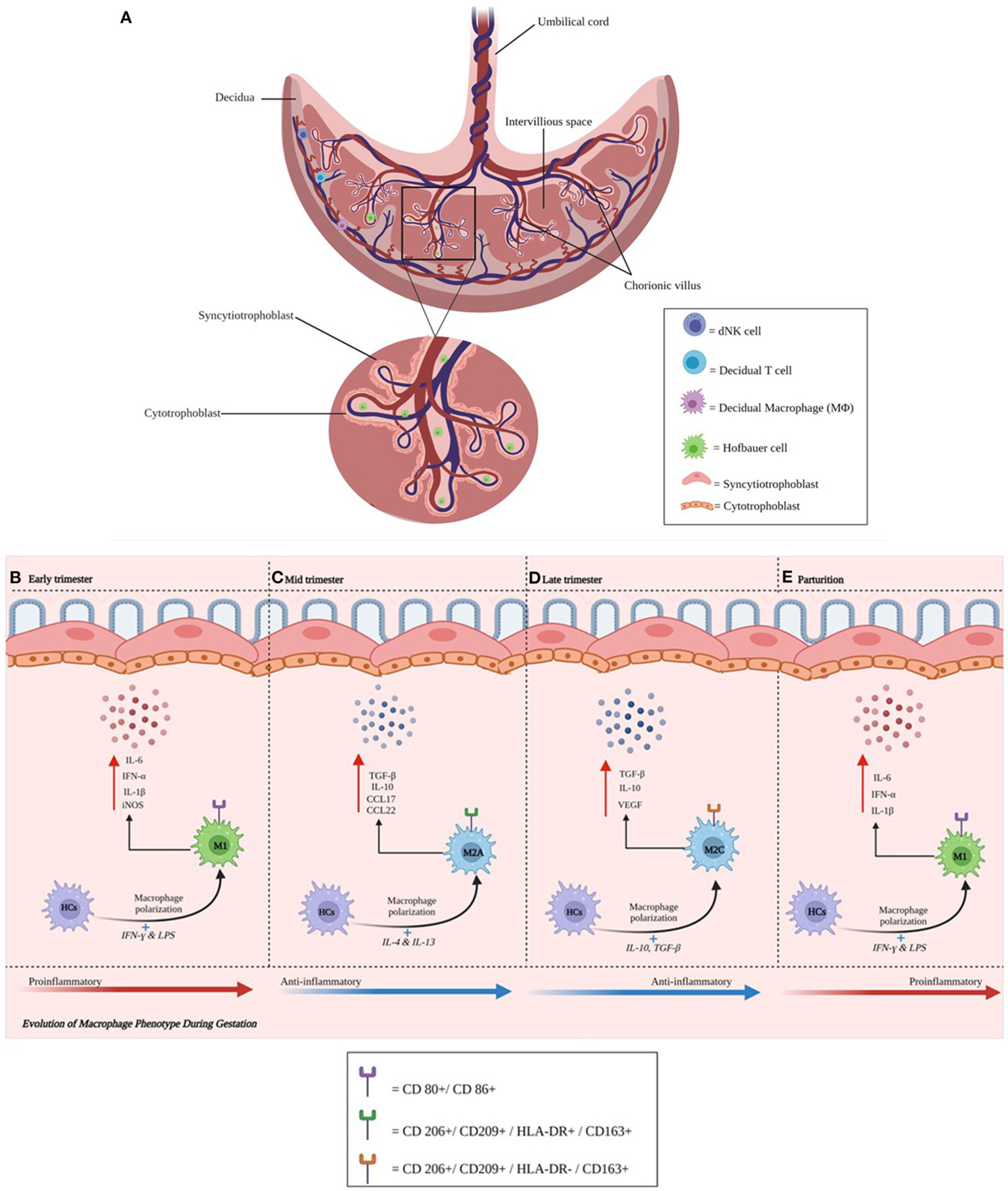
**(A–E)** Anatomical illustration of the placenta. The placenta plays a critical role in a successful pregnancy and fetal development. Chorionic villi (magnified) support contact with the maternal circulation. Individual cells and their respective locations in the decidua (natural killer, T, and macrophage cells) and villi (Hofbauer cells) are illustrated. Cytotrophoblast cells are found in the decidua basalis and in placental villi, while syncytiotrophoblast cells line placental villi. This interface facilitates delivery of nutrients and oxygen from endometrial arteries. During maternal infection, pathogens may breach the trophoblast layer and migrate toward the fetal side of the interface. HCs change their phenotype during gestation. Activated HCs are abundant in early pregnancy and become regulatory thereafter until parturition. **(B–E)** Represents the phenotypic changes that Hofbauer cells undergo during gestation.

Many of the studies addressing viral infection of the placenta have focused on characterizing the presence and role of decidual natural killer (dNK) cells and macrophages, trophoblasts including extra villous trophoblasts (EVTs), T cells in the decidua and villi, and placental macrophages or Hofbauer cells (HCs) located in underlying stromal tissue (Figures 1A–E). It is important to elucidate mechanisms that HIV-1, HCMV, and ZIKV utilize to gain access to the fetal side, characterize their intracellular effects, and identify correlates of protection. We will therefore focus on current knowledge on individual host cell-viral interactions within these immune populations in the decidua and placenta. The significance of characterizing placental immune cell-viral interactions is underscored by the fact that infectious complications that directly impact the placenta lead to adverse outcomes in both mother and fetus/newborn, contributing significantly to perinatal morbidity and mortality, and is further highlighted by effective antiviral and immunomodulatory therapies that can improve clinical outcomes in the dyad.

The known underlying mechanisms of pathogenesis, protection, and transmission at the MFI to HIV-1, HCMV, and ZIKV are discussed in this article following summaries on embryology and placental immunity. In general, most of the articles cited reflect human studies unless otherwise stated. Discrepancies have arisen when comparing human with murine or other animal placentae. Identifying an ideal animal model system for congenital HCMV infection has remained a significant challenge. At the end of each section, current knowledge gaps that may prompt future research directions are also discussed.

### Embryology

Three main placental types are recognized according to the cell layers comprising the interhemal area: (1) epitheliochorial type (horses, pigs and ruminants), (2) endotheliochorial type (carnivores) and (3) hemochorial type (primates, rodents and rabbits). The number of cell layers and placental permeability in the interhemal area modifies the exchange of nutrients between maternal and fetal blood and is one of the important factors differentiating mammalian species (3). The single-celled embryo resulting from fertilization undergoes successive divisions to derive a blastocyst, which consists of two cell types: the outer trophoblast or trophoectoderm (TE) layer forming the placenta and chorion, and the inner cell mass developing into the embryo proper and amnion. Embryonal implantation varies widely in eutherian mammals and can be distinguished by the degree of invasion of the blastocyst into the endometrium. There are two types of attachment: superficial and interstitial. The latter is found in rodents and primates (4). The TE gives rise to cytotrophoblast (CTB) cells, which follow villous and extravillous pathways. In the former, mononuclear CTBs fuse, creating multinucleated syncytiotrophoblasts (STBs) (5). The STB, specialized for exchange of nutrients and waste products between maternal and fetal compartments, expresses the neonatal Fc receptor (FcRn), which binds maternal IgG and transcytoses this immunoglobulin for passive immunity (6, 7). During human placentation the stromal compartment of the endometrium differentiates into the decidua enabling villous trophoblasts of fetal origin to invade this compartment and the inner third of the myometrium. Thereafter, the trophoblast differentiates and fetal EVTs penetrate and remodel uterine arteries (8). This invasion occurs in the presence of immune cells, so that the branched villous tree-like structures which form, contain fibroblasts, immature capillaries, along with dNK and T cells (9) (Figures 1A–E). Gaining a better understanding of the changes that occur during normal gestation may provide invaluable insights into pregnancy-associated conditions that have an immunological basis, such as preterm labor and villitis of unknown etiology (VUE). New findings could promote the development of strategies to improve perinatal outcomes for both mother and baby. Areas of future investigation include characterization of genes regulating immune cells at the MFI and identification of signaling pathways between the embryo and uterus that impact the regulation and trafficking of dNK and T cells, and macrophages, during normal pregnancy and those affected by maternal infection.

### Human Placental Macrophages

Placental fetal macrophages or HCs change their phenotype during gestation (Figures 1B–D). We have previously shown that activated HCs are abundant in early pregnancy and decreased by term and their molecular signatures emphasize an inflammatory phenotype early and in very late gestation. The frequency of HCs with a regulatory phenotype remain high through term although term HCs exhibit blunted responses to stimulation (Figures 1B–D). Ligand-specific responses are temporally regulated: we noted an absence of inflammatory mediators and reduced antiviral gene transcription following retinoic acid-inducible gene I (RIG-I) activation at term, despite these cells producing inflammatory mediators following interferon–γ (IFN-γ) plus lipopolysaccharide (LPS) stimulation (10). Other groups have recently shown that early-trimester HCs, which are transcriptionally similar to yolk sac macrophages, exhibit microbicidal activity including expressing toll-like receptor (TLR)-6 (which binds to bacterial lipoproteins), and have a distinct response to TLR-6 stimulation (11). Collectively, these studies suggest HCs play key roles in protective immunity as well as fetal tolerance throughout gestation. In contrast, decidual macrophages may have an immunoregulatory phenotype expressing CD206 and DC-SIGN, associated with M2 polarization. The recent publication by Thomas et al. using a novel flow cytometric gating strategy characterized first-trimester human HCs as HLA-DR^−^ folate receptor 2 (FOL2)^+^. The group also identified a newly characterized population of placenta-associated maternal macrophages (PAMM1a) that may be involved in tissue repair (11). These cells and their regulation and functional roles need to be more extensively characterized. Future research should address the phenotype and function of HCs and decidual macrophages *ex vivo* across gestation and their role in health and during maternal infection.

### Decidual NK Cells

NK cells are a major component of innate immunity. The decidua is made up of 40% leucocytes of which 20% are macrophages and 70% tissue resident dNK cells during the first trimester of pregnancy. Decidual NK cells exhibit a phenotype that contrasts with their peripheral counterparts and are CD56^superbright^ CD16^neg^ constitutively expressing the activation marker CD69. Decidual NK cells can produce large amounts of cytokines, chemokines and angiogenic factors, and gain cytotoxic function in the presence of specific pathogens (12, 13). Human dNK cells were recently shown to express the antimicrobial peptide granulysin and selectively transfer this peptide via nanotubes to EVTs to kill intracellular *Listeria monocytogenes* without killing the trophoblast (14).

The discovery of three main pools of dNK cells (dNK1, dNK2, and dNK3) by single-cell RNA sequencing (scRNAseq) and profiling the transcriptomes of ~70,000 cells from first-trimester placentae with matched maternal blood and decidual cells by Vento-Tormo et al. (15) identified different immunomodulatory profiles that may be important in preventing detrimental immune responses at the MFI. Despite the elegant characterization, the exact origin and function of the three dNK cell subsets require further study. Vieira et al. recently showed that (i) term dNK cells have an increased degranulation response to K562 and PMA/Ionomycin (PMA/I) but lower capacity to respond to HCMV-infected cells; (ii) term pregnancy dNK cells do not recognize HLA-C, as has been shown for first trimester dNK cells; (iii) protein and gene expression profiles identified multiple differences between peripheral NK cells, first trimester, and term pregnancy dNK cells, suggesting the latter are phenotypically and functionally distinct (16). Areas of further investigation could therefore include elucidating the potential role of “trained” or memory dNK cell immunity and how these cells restrict viral replication at the MFI (13). It will also be important to determine the relative contribution of innate intrinsic factors in the placenta that contribute to viral restriction.

### Placental T Cells

The decidual CD4+ population is comprised of about 50% activated memory CD25^dim^ T cells and 5% CD4+ CD25^bright^ FOXP3+ Treg cells. In addition, ~40% of decidual CD8+ cells have an effector-memory phenotype with reduced perforin and granzyme B release compared to peripheral CD8+ T cells (17). A recent study from our group examining placentae from women with HIV showed inverted CD4:CD8 ratios and higher proportions of tissue resident CD8+ T cells in villous tissue relative to control placentae. CD8+ T cells in the fetal capillaries, which were of fetal origin, positively correlated with maternal plasma viremia prior to antiretroviral therapy (ART) initiation, suggesting imbalanced T cells persisted throughout pregnancy. Additionally, the expanded memory differentiation of CD8+ T cells was confined to the fetal placental compartment and cord blood but not detected in maternal decidua (18). These intriguing findings require further corroboration on flow cytometry and transcriptomic and proteomic platforms. Our immediate questions include what prompted migration of fetal CD8+ T cells into placental villi? Why did these cells exhibit expanded or late memory differentiation? Do these CD8+ T cells exhibit specificity against HCMV or HIV-1? Are these cells protective or does potential release of inflammatory granzyme B and perforin promote villitis and vertical transmission of HIV-1?

Chronic villitis is an inflammatory condition of third trimester placentae characterized by infiltration of maternal CD8+ T cells into villi, which may either reflect an infection or a tissue rejection response to the haploidentical fetus [i.e., villitis of unknown etiology (VUE)]. It is important to note that chronic villitis, specifically VUE, has been observed in placentae from term infants with normal outcomes as well as in infants born following complicated gestations (19). There remains a paucity of data on the mechanisms controlling these outcomes. We therefore characterized the global TCR β-chain profile in human T cells isolated from placentae diagnosed with VUE at term. We compared cells from controls and from placentae with infectious villitis using immunoSEQ. We demonstrated that VUE is driven predominantly by maternal T cell infiltration; however, these abundant T cell clones showed very little overlap between subjects. Mapping TCR clones to common viral epitopes (for HCMV, Epstein-Barr Virus, and influenza A) demonstrated that antigen specificity in VUE was equal to controls and significantly lower than HCMV-specific clones in infectious villitis, indicating VUE represents an allograft response, not an undetected infection (20). The immune phenotype of HCMV-specific T cells of fetal origin identified in placental villi from infants who were diagnosed with congenital HCMV infection requires further characterization. This includes determination of specific immune function and their potential role in viral transmission or protection.

Against this background and after summarizing clinical presentations and epidemiology, the rest of this article will discuss the role of decidual macrophages and NK cells, and then focus on trophoblasts (including EVTs), HCs and T cells, and their relationship with HIV-1, HCMV, and ZIKV. We will address how each virus as mono- or co-infection (HIV-1 and HCMV) interacts with decidual and placental immune cells elucidating mechanisms of viral entry to the fetal side, characterizing their intracellular effects, and discuss potential mechanisms of protection.

## VIRAL PATHOGENS

### Endogenous Retroviruses and Syncitins at the Maternal-Fetal Interface

Mechanisms of protection have evolved at the MFI over millennia to restrict retroviral infection following exposure. Endogenous retroviruses (ERVs) are molecular remnants of ancient retroviruses that point to an ongoing relationship between lentiviruses and vertebrate hosts that began hundreds of millions of years ago. Retroviruses may have acted on vertebrates as agents of selection, driving the evolution of host genes that block viral infection. Furthermore, through a process of endogenization, retroviruses have contributed an abundance of genetic novelty to host genomes, including unique protein-coding genes and cis-acting regulatory elements. In the placenta, syncytins are glycoproteins of retroviral origin that fulfill cellular functions involving receptor-mediated membrane fusion, promoting merging of CTBs to form the multinucleate STB layer. Their potential role in HIV-1 transmission is discussed below (21).

## HIV-1

### Viral Tropism

In general, the primary target for HIV-1 are thought to be activated memory CD4+ T cell populations, but many other cell types, including naïve CD4+ T cells, macrophages, dendritic cells, and monocytes can also be infected (22, 23). The primary host molecule targeted by HIV-1 is the CD4 receptor, with chemokine receptors CCR5 and CXCR4 serving as co-receptors. The HIV-1 glycoprotein gp120, present on the viral envelope, is responsible for binding CD4 and either CCR5 or CXCR4 to initiate membrane fusion and cell entry (24).

### Interventions to Prevent Vertical Transmission During Maternal HIV-1 Infection in Pregnancy

Globally, over 60 million people have been infected by HIV-1. Maternal HIV-1 infection during pregnancy is associated with vertical or mother-to-child transmission (MTCT). MTCT of HIV-1 occurs most often intrapartum, during labor and delivery, and then through breastfeeding. In the absence of clinical interventions, the risk of intrauterine transmission is estimated at 5–10%. Current United States (US) guidelines from the Department of Health and Human Services recommend administration of ART throughout pregnancy to maintain maternal plasma viral concentrations beneath the level of detection and the avoidance of breast feeding (25). Other interventions include Cesarean section and intrapartum prophylaxis with intravenous Zidovudine, depending on the clinical circumstances. These recommendations have dramatically reduced the overall risk of MTCT of HIV-1 (intrauterine, intrapartum, and post-partum through breast feeding) to 1–2% from >25% before the evidence base for these guidelines began to emerge (26).

### Genital Neutrophils in HIV-1-Exposed Women

In order to develop strategies for prevention of MTCT of HIV-1, it is important to characterize target cells in the female reproductive tract mucosae and identify effective innate responses. Women acquire HIV-1 mainly through sexual intercourse. However, low transmission rates per sexual act suggests local immune mechanisms contribute to prevention. Neutrophils mediate mucosal protection against bacterial and fungal pathogens including by release of neutrophil extracellular traps (NETs). These DNA fragments associate with antimicrobial granular proteins. Stimulation of human genital neutrophils with HIV-1-viral-like particles (HIV-1-VLPs) induced NET release following viral exposure, through reactive oxygen species-independent mechanisms resulting in entrapment of HIV-1-VLPs. Incubation of infectious HIV-1 with pre-formed genital NETs prevented infection of susceptible cells through irreversible viral inactivation. HIV-1 inactivation by NETs from genital neutrophils may represent a previously unrecognized form of mucosal protection against viral acquisition (27).

### HIV Interactions With Human Hofbauer Cells

The specific mechanisms that offset intrauterine transmission during HIV-1 infection in pregnancy are poorly characterized. Intriguingly, substantial numbers of maternal cells appear to cross the placenta and reside in fetal lymph nodes, inducing development of CD4+CD25^high^FoxP3+ T regulatory cells (28). By extrapolation, during maternal HIV-1 infection (in the absence of ART administration), HIV-1-infected immune cells transiting the placenta to the fetus should facilitate intrauterine MTCT with rates closer to 100% rather than 5–10%. Just as perplexing is the observation that placental HCs, which are present in large numbers throughout gestation in placental stroma and express all the receptors needed to facilitate and promote HIV-1 transmission and dissemination, namely CD4, CCR5, CXCR4, and Dendritic Cell-Specific Intercellular adhesion molecule-3-Grabbing Non-integrin (DC-SIGN) or CD209, seemingly protect against rather than facilitate MTCT of HIV-1 (29). Areas of further research include determining how fetal immunity confers relative resistance to MTCT of HIV-1 and identifying signals that prompt phenotypic changes in fetal immune cells to a more immunoreactive adult phenotype (8).

**FIGURE 2 |:**
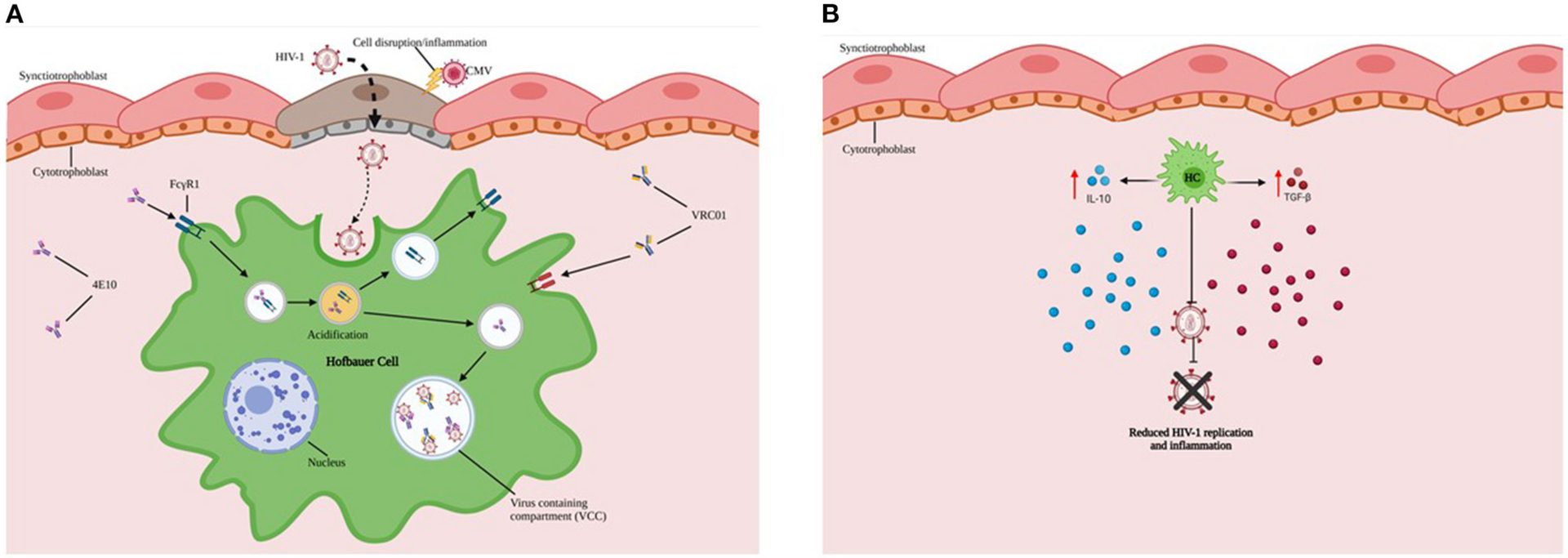
HCs exhibit reduced ability to replicate HIV-1. **(A)** HCs (Green) sequester virus and may serve as protective reservoirs to permit intracellular neutralization in virus-containing compartments (VCCs in Blue). **(B)** Endogenous production and secretion of immunoregulatory cytokines such as IL-10 and TGF-β by HCs are important in not only supporting tolerance to the haploidentical fetus but also limiting inflammation and viral replication during maternal HIV-1 infection.

Over the last decade, our group and other investigators have characterized innate and adaptive mechanisms of immune protection against HIV-1 at the MFI that may contribute to how the placenta prevents vertical transmission of HIV-1 during gestation (Figures 2A,B) (25, 29–32). Early in the HIV-1 pandemic, investigators applied immunohistochemistry to visualize HIV-1 antigens on the fetal side of the placenta at 8 weeks gestation (33). The low observed rate of intrauterine transmission (5–10%) likely reflect innate and adaptive barriers to fetal acquisition of HIV-1.

Major findings from our research include: 1) HCs exhibit reduced ability to replicate HIV-1 *in vitro*; 2) HCs sequester virus and may permit intracellular neutralization in virus-containing compartments (Figure 2A) and ARV drug entry; 3) While HCs are susceptible to HIV-1 infection and replicating virions can be identified in virus-containing compartments, the transcriptional and replication kinetics of HIV-1 in HCs are slow compared to monocyte-derived macrophages (MDMs) (34). 4) In addition, HCs constitutively express high levels of immunomodulatory cytokines (including interleukin (IL)-10 and Transforming Growth Factor (TGF-β), which may restrict HIV-1 replication (Figure 2B) (29). 5) Limited availability of CD4+CCR5+ target cells in cord blood may restrict establishment of viral infection.

Most recently we have also shown that HCs early in gestation express higher levels of CCR5 and exhibit a more activated phenotype yet are less permissive to HIV-1 replication than term HCs with downregulated CCR5 levels. This observation may reflect that early/mid gestation HCs display overall increased production of anti-inflammatory cytokines, chemokines, and an IFN-dependent antiviral immune response. In contrast, term HCs exhibit attenuated IFN-induced Signal Transducer And Activator Of Transcription (STAT)1 and STAT2 activation. Treatment of early/mid gestation and term HCs, with type I IFNs or RIG-I-like receptor (RLR) agonists reduced HIV-1 replication in both populations, underscoring the importance of IFN and RLR signaling in inducing an antiviral state. Viral recognition and antiviral responses in early gestation HCs may prevent intrauterine HIV-1 infection, whereas diminished antiviral responses at term may facilitate MTCT of HIV-1 (32).

### Fetal HCMV Infection Promotes Intrauterine HIV-1 Transmission—The Role of Co-infection

Along with HIV-1, HCMV is the most common viral agent transmitted from mother to child and may contribute to vertical transmission of HIV-1. There is emerging clinical (intrauterine and intrapartum) and *in vitro* data associating HCMV with MTCT of HIV-1 (31, 35, 36). However, the mechanisms that promote transmission are poorly characterized. A study examining the timing of HCMV infection relative to HIV-1 infection found that fetal HCMV infection correlated with both *in utero* and intrapartum HIV-1 infection (37). We previously demonstrated that HCMV coinfection enhances susceptibility and viral replication of HIV-1 in HCs (30). Consistent with enhanced viral susceptibility, HCMV exposure upregulated CCR5 and CD80 expression on HCs and also significantly induced type I IFN, proinflammatory cytokines, and antiviral gene expression. Interestingly, we found that HCMV diminished type I IFN-mediated phosphorylation of STAT2.

### Intrauterine Transmission of HIV-1—A Role for Cytotrophoblasts?

**FIGURE 3 |:**
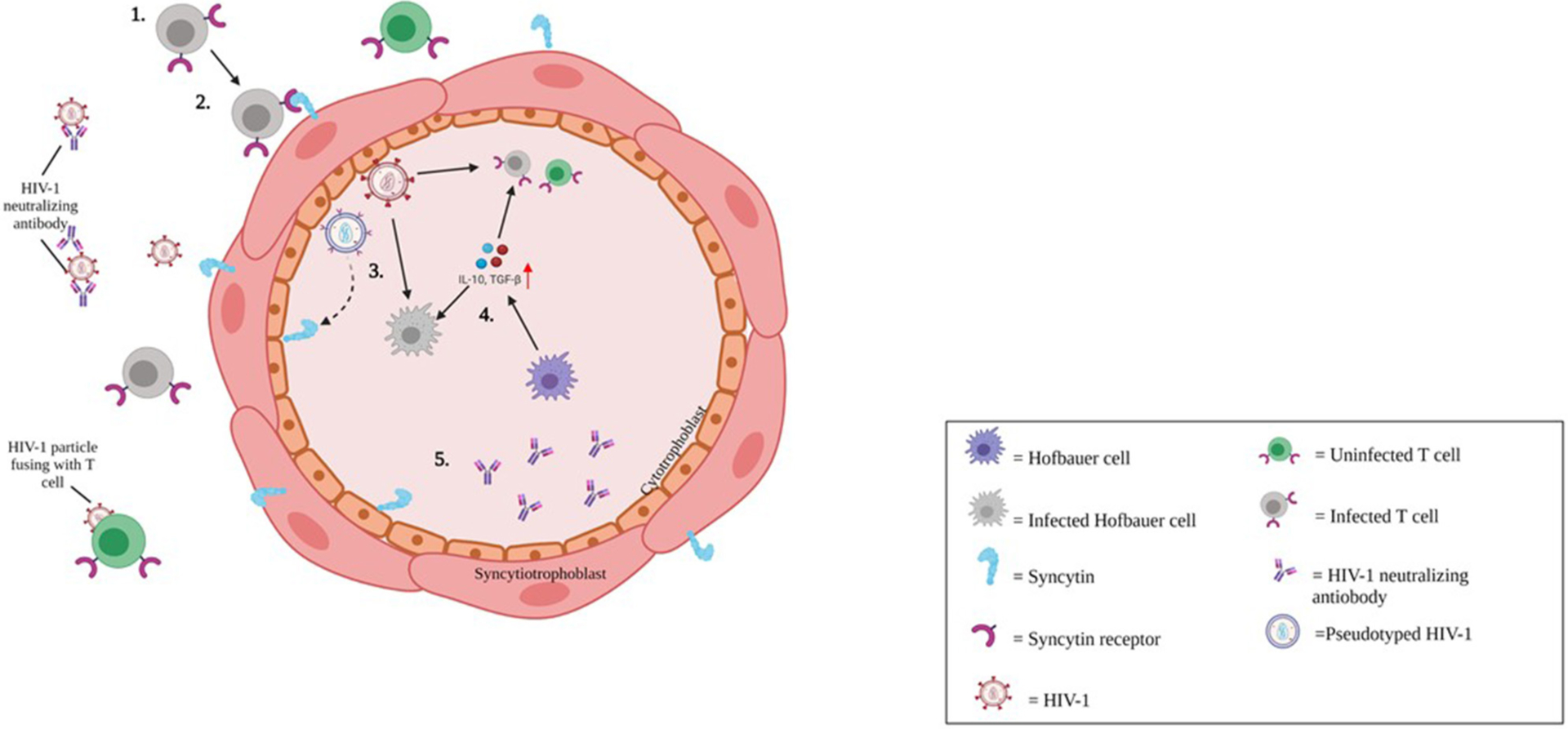
Putative mechanism of HIV-1 infection of cytotrophoblasts. 1) Cell-associated HIV-1 in the maternal circulation either antibody coated, or antibody free reach the maternal-fetal interface of the placenta. 2) HIV-1 infection is restricted to CD4+ CCR5+ tropic cells. However, endogenous retroviral fusion protein, syncytin expressed on trophoblast cells may facilitate entry of HIV-1 via membrane fusion. 3) HIV-1 infection in cytotrophoblasts (CTBs) may lead to production of pseudotyped HIV-1 (Blue virion), and CD4+ tropic HIV-1 (Red virion). 4) HIV-1 transmission to the fetus may be offset by CTBs and innate antiviral responses. Hofbauer cells (HCs; Purple cell) may be targeted by HIV-1 infection (gray indicates HIV-1-infected HC).

Cytotrophoblasts (CTBs) have not been documented as target cells for HIV-1 in the scientific literature, although Tang et al. recently described a potential route of CTB infection by cell-to-cell fusion. They detected HIV-1 RNA and cDNA in placental tissue, including CTBs from pregnant HIV-1-infected women receiving ART. They suggested syncytin, the envelope glycoprotein of human ERV family W1 expressed on CTBs, triggered membrane fusion with syncytin receptors present on HIV-1-infected CD4+ T cells, leading to production of two different types of progeny HIV-1 virion (Figure 3) (38).

### The Role of Decidual Cells in Limiting HIV-1 Replication

Decidual NK cells may play a role in limiting HIV-1 infection in decidual macrophages (dM) at the MFI during the first trimester. dNK cells constitute nearly 70% of the total decidual leukocyte population (13). Peripheral or conventional NK cells (cNK cells) control HIV-1-infected cells, either directly by either contact-mediated or antibody-dependent cellular toxicity. An additional factor that may regulate HIV-1 infection in the first trimester is dNK secretion of IFN-γ and chemokines (CCL3 and CCL4), which inhibit HIV-1 infection of monocyte-derived dendritic and mononuclear cells, facilitated by cell-to-cell contact between dM and dNK cells to achieve optimal inhibition. Quillay et al. have shown that dNK cells control HIV-1 infection of dMs *in vitro* through cellular contact and release of soluble factors with IFN-γ involved in this control (39, 40). Given the anti-HIV-1 properties of dNK cells, one promising intervention to treat or even cure HIV-1 infection is the use of chimeric-antigen-receptor (CAR) NK cells cultured to target HIV-1 gp160 on the membrane of infected target cells (41).

### Adverse Clinical Outcomes in HIV-1-Exposed Uninfected Children

A number of investigators have noted that even when MTCT of HIV-1 is prevented, HIV-1-exposed infants and children (HEU) exhibit impaired immunity in postnatal life associated with adverse clinical outcomes compared with HIV-unexposed/-uninfected (HUU) children. Most infection-related hospitalizations are associated with bloodstream or respiratory infections (17, 42, 43). HCMV infection in HIV-1-infected pregnant women was shown to alter fetal immune responses even in the absence of MTCT of HIV-1 (44). More recently, Pereira et al. noted that innate immune factors such as high mobility group box protein 1 (HMGB1), IL-1, and type I and III IFNs, as well as their signaling molecules IFN-regulatory factor 3 (IRF3) and IRF7, were upregulated in placental villi from women with HIV-1 infection. However, these innate immune factors were simultaneously decreased in maternal serum, indicating that pregnancy may restrict immune activation systemically in the mother, while permitting or inducing an antiviral state locally within placental cells (17). With the global HEU infant population growing rapidly (>1.5 million), the public health importance of identifying modifiable biological mechanisms at the MFI contributing to health disparities between HEU and HUU children is a global health priority.

## HCMV

### HCMV Tropism

HCMV has a wide range of tropism and can infect epithelial, myeloid, and endothelial cells. However, the mechanisms of fetal CMV infection are not yet fully delineated (45), and animal models do not faithfully recapitulate MTCT in humans. Investigators have therefore often characterized HCMV in primary human placental cells and explant models (46). We previously demonstrated HCMV infection of human CTBs and HCs (47, 48).

### The Epidemiology and Clinical Characteristics of Congenital HCMV

Globally, many individuals are infected with HCMV reflecting the ubiquity of this virus, ability to spread by horizontal and vertical transmission, and to manifest with subclinical illness for nearly all individuals including pregnant women. An HCMV seroprevalence of 100% is estimated in many parts of Africa and Asia with numbers up to 86 % in women of childbearing age. The lowest seroprevalence is seen in the WHO European region at 66% (49, 50). HCMV is the most common congenital infection worldwide, and in the US, estimated to occur in 1 out of 200 live births. Human CMV establishes lifelong latency following primary infection. Maternal primary infection during early gestation (first or early second trimester) is associated with an increased incidence of fetal HCMV infection (~40%) compared to reactivation from latency (<1%). HCMV transmission to the fetus can be categorized into (1) transplacental, (2) intrapartum, and (3) breast milk. Congenital HCMV infection can be asymptomatic, but severe manifestations include sensorineural hearing loss, intrauterine growth restriction, rash, cognitive delay and impairment of neurodevelopment, chorioretinitis, cerebral abnormalities including periventricular calcifications, and hepatosplenomegaly (45, 50–52). HCMV accounts for 25% of all cases of sensorineural hearing loss in children (53, 54). There are a paucity of animal models characterizing HCMV in the mother-infant dyad, although Almishaal et al. (55) recently utilized a mouse model to recapitulate features of congenital HCMV-mediated childhood hearing loss, demonstrating that NK cells play a protective role.

HCMV is one of 8 human herpesviruses that can infect humans at all ages. Once born, young children are frequently exposed to HCMV in communal settings and the Centers for Disease Control and Prevention (CDC) and WHO estimate that ~30% of children in the US are HCMV seropositive by age five (56, 57). Commercially available vaccines against herpesviruses are limited to varicella-zoster virus (VZV), the etiological agent for chickenpox/shingles. A vaccine against HCMV is not currently available.

Innate and adaptive placental immunity may promote viral entry into placental villi and cord blood. In the following sections, we will discuss viral entry in and the intracellular effects of HCMV infection in immune placental cells, which may lead to adverse birth outcomes.

### Mechanisms of HCMV Entry at the MFI

**FIGURE 4 |:**
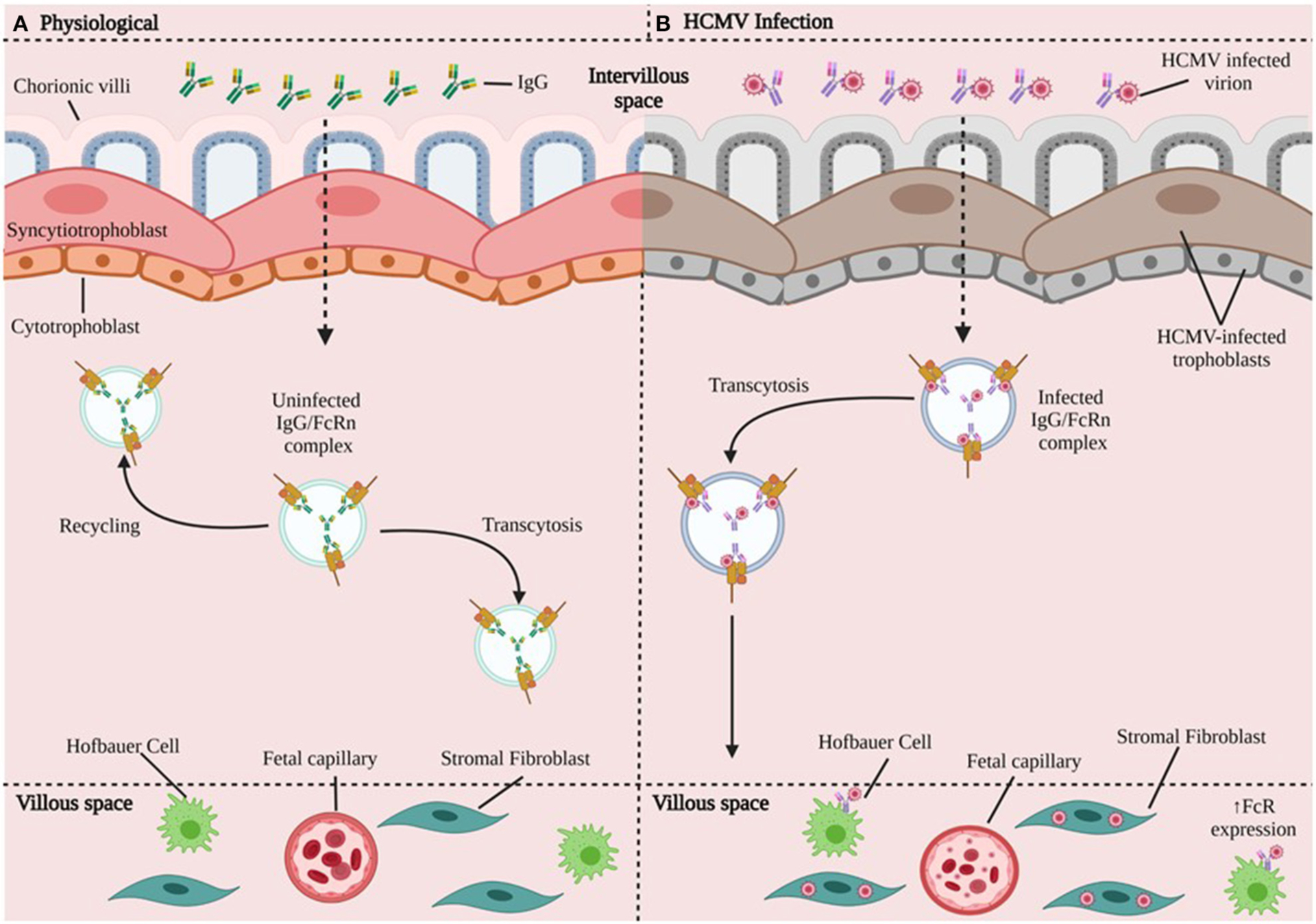
HCMV transmission in chorionic villi. **(A)** In the physiologic state, maternal IgG binds FcRn in endosomes and can take different paths: either recycle (going back to the surface) or undergo transcytosis toward the basal membrane and bind to cells that express FcR. **(B)** HCMV Infection. The HCMV-infected virion disrupts trophoblast cells and forms HCMV IgG immune complexes that undergo FcR-mediated transcytosis. The HCMV-IgG immune complex can go on to infect HCs, fibroblasts, fetal capillaries. HCMV may be able to increase the expression of FcR (59). CTBs are important sites for placental HCMV infection, most likely transmit virus to the fetus, and HCMV could disseminate to the placenta by co-opting the receptor-mediated transport pathway for IgG.

Applying immunohistochemical analysis of early gestation fixed villous explants and placental biopsy specimens, Maidji et al. demonstrated HCMV replication proteins in villous CTBs, while STBs were spared. This pattern suggested virion transcytosis across the surface. In contrast, STBs from placentae with high neutralizing titers contained viral DNA and caveolin-1-positive vesicles in which IgG and HCMV glycoprotein B co-localized. Quantitative analysis in polarized epithelial cells showed that FcRn-mediated transcytosis was blocked by the Fc fragment of IgG, but not F(ab^′^)_2_. These results suggest CTBs are important sites for placental HCMV infection, most likely transmit virus to the fetus, and that HCMV virions could disseminate to the placenta by co-opting the receptor-mediated transport pathway for IgG (58) (Figure 4).

More recently, Naing et al. demonstrated that HCMV uses different entry pathways, involving at least the viral pentameric complex gH/gL/pUL128-pUL131A, and cellular platelet-derived growth factor receptor-α (PDGFRα) for entry into first trimester extravillous-derived (SGHPL-4) and villous-derived (HTR-8/SVneo) trophoblasts. Infection with four HCMV clinical and laboratory strains (Merlin, TB40E, Towne, AD169), and Merlin deletion mutants of UL128-, UL130-, and UL131A-genes, showed a cell type-dependent requirement of the viral pentameric complex for infection of trophoblasts. The viral pentameric complex was essential for infection of villous trophoblasts, but not for EVTs. Blocking of PDGFRα in EVTs, which naturally express PDGFRα, inhibited entry of pentameric complex-deficient HCMV strains, but not the entry of pentameric positive HCMV strains (60).

### Decidual and Placental Macrophages During Maternal HCMV Infection

We have previously demonstrated HCMV infection of term human CTBs and HCs when testing whether Modified Vaccinia Ankara (MVA)-gH-gL-pentamer complex vaccine induced neutralization titers in sera from vaccinated Rhesus Macaques to block HCMV TB40/E infection of freshly isolated HCs and CTBs. HCMV entry into both cell types is blocked by gH/gL-PC-specific neutralizing antibody (47, 48). However, the role of HCs and decidual macrophages in the transmission and dissemination of HCMV to surrounding tissue in the placenta and decidua, and how HCMV infection of these cells results in impairment of placental development and function is largely unknown.

### Decidual and Placental T Cells During Maternal HCMV Infection

During HCMV infection in pregnancy, naïve CD8+ T-cells (CCR7+CD45RA+) were reduced by 50% in maternal blood. However, there was a doubling in the proportion of CD45RA+ revertant memory cells (CCR7-CD45RA+) in seropositive donors. Moreover, seropositive women during late pregnancy demonstrated an accumulation of highly differentiated HCMV-specific T-cells (61). There are few descriptions identifying T cells during HCMV infection at the MFI. As previously discussed, we have detected tissue resident CD8+ T cells in villous tissue from women with HIV-1 infection relative to control placentae. CD8+ T cells in the fetal capillaries, which were of fetal origin and exhibited expanded memory differentiation, persisted throughout pregnancy (18).

**FIGURE 5 |:**
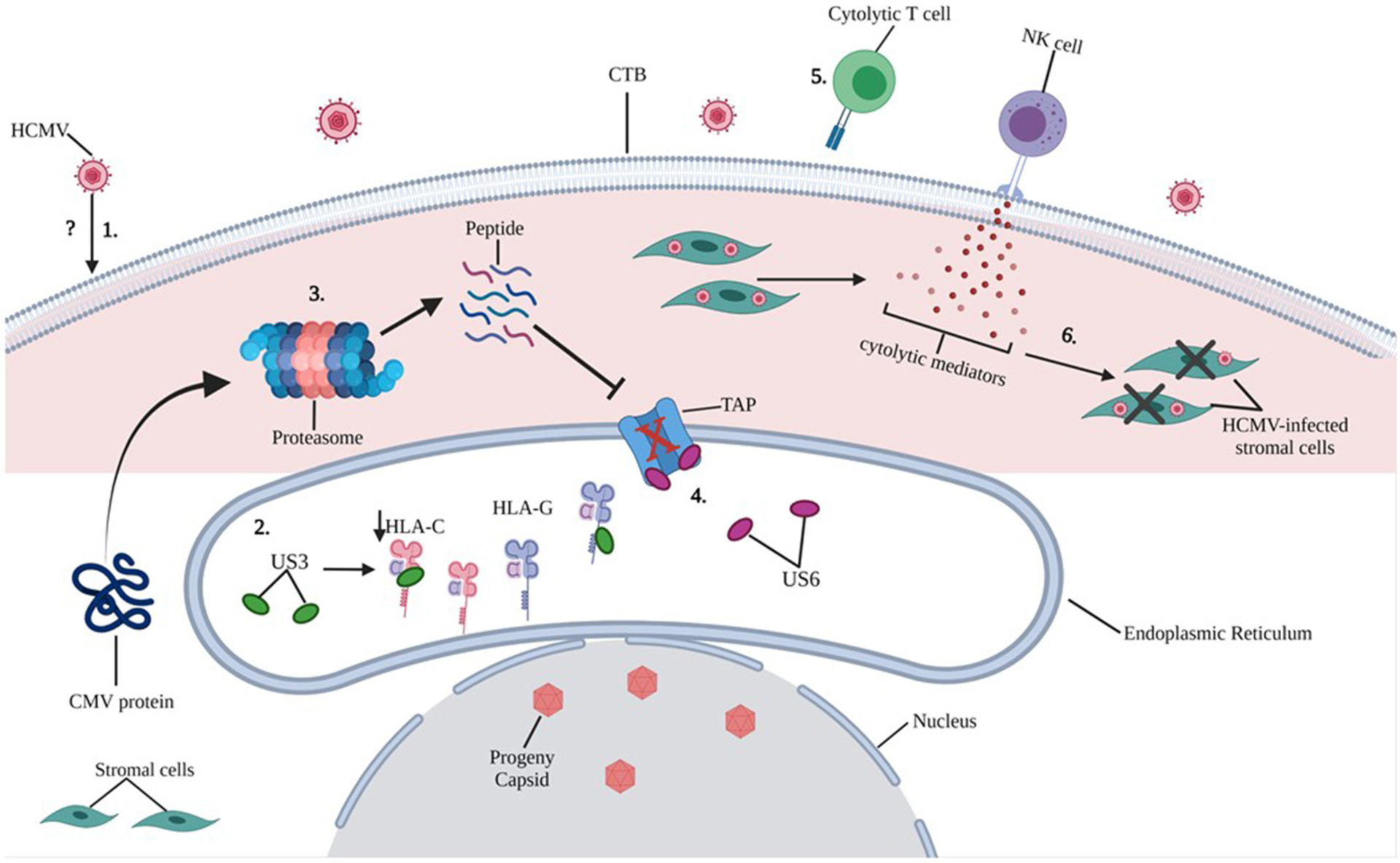
Putative mechanisms of HCMV induced intracellular damage in trophoblast cells. 1) The exact mechanisms by which HCMV infects trophoblast cells remain unclear (refer to Figure 4), however, molecular mechanisms by which HCMV causes intracellular damage are well documented. 2) HCMV glycoprotein US3 directly binds to both HLA-C and HLA-G within the endoplasmic reticulum (ER) and blocks transportation of MHC molecules only partially downregulating HLA-C upon HCMV infection of primary EVT cells. 3) Cytoplasmic proteins are constantly monitored by proteosome processing and peptides are retrograde transported to the ER through transporter TAP to be loaded onto the MHC. 4) HCMV glycoprotein US6 inhibits the TAP, and the cytoplasmic peptide-including HCMV derived peptide are unable to enter the ER. 5) Because of reduced expression of MHC class I molecules on HCMV-infected CTBs, cytolytic T cell recognition is impaired. 6) NK cell-mediated cytolytic killing of HCMV-infected stromal cells may result in placental damage.

Extravillous trophoblasts (EVTs) appear to escape CD8+ T cell recognition through a lack of expression of major histocompatibility complex (MHC-I) receptors: HLA-A and HLA-B, which promote cytotoxic activity. CTBs express both classical (HLA-C) and non-classical (HLA-G) class I MHC-I receptors. Interestingly, HCMV did not induce a significant difference in HLA-G expression on either JEG-3 cells or primary EVTs, and only partially downregulated HLA-C. Persistence of HLA-G expression, which is associated with immune tolerance at the MFI despite HCMV infection, suggests protection of infected trophoblast cells from the effects of CD8+T cells. In this same article, however, dNK cells were able to kill HCMV-infected maternal stromal cells (62) (Figure 5).

### Decidual NK Cells During Maternal HCMV Infection

NK cell diversity is based on NK receptor expression, distinct NK cell types and differentiation states, which accommodate a variety of dNK cell functions at the MFI. dNK cell antiviral activity and immunity to HCMV infection occurs in tandem with T cell activation, resulting in secretion of cytokines and interaction with EVTs. These functions may change depending on gestational age and decidual tissue type. As discussed in the previous section, first trimester dNK cells were able to clear HCMV-infected cells but failed to increase degranulation and cytokine production in response to HCMV-infected EVT cells (62). Vieira et al. (16) were then able to show that these cytotoxic responses in the first trimester were distinct compared to responses at term, which exhibited reduced efficacy in responding to HCMV-infected cells. Cell death may occur through formation of immunological synapses with HCMV-infected fibroblasts, enabling the delivery of perforin/granzyme for cellular destruction and by lytic granule (including granulysin) release (63). Crespo et al. were also able to show that activating dNK cell receptors including KIR2DS1, KIR2DS2, KIR2DS5 and KIR3DS1 correlated with antiviral activity and cytotoxicity. Specifically, that KIR2DS1 positive dNK cells showed increased cytotoxicity to HCMV-infected decidual stromal cells (DSCs) positive for HLA-C2 when compared to KIR2DS1negative dNK cells (62).

The main surface marker characterizing a specific NK cell subset during HCMV infection is the activating receptor NKG2C, a C-type lectin that recognizes HLA-E and frequently dominates HCMV-mediated expansion (64). A higher proportion of NKG2C^+^ NK cells after HCMV infection have been detected in children with symptomatic congenital HCMV infection (65). A unique population of dNK cells, characterized by increased NKG2C and LILRB1 expression, was recently detected in multigravid women. These cells are mostly confined to the NKG2C^hi^ decidual “memory” NK phenotype, and have been termed Pregnancy Trained decidual NK cells (PTdNKs) (66). The function of PTdNKs during primary maternal HCMV infection and reactivation and during other viral infections in pregnancy requires further elucidation.

### Mechanisms of HCMV-Induced Damage at the MFI

In normal early gestation, CTBs secrete matrix metalloproteinase-9 (MMP-9), which may enable cells to digest extracellular matrix to complete differentiation and invasion. MMP-9 levels gradually decrease during pregnancy. Once differentiation and invasion are complete, CTBs secrete IL-10 to modulate the immune response to protect the fetus. HCMV infection at the MFI can downregulate MMP-9 activity, so that HCMV-infected CTBs induce HCMV-IL-10 mRNA release. HCMV-IL-10 mRNA in turn, may acts as an agonist at the IL10-receptor, reducing MMP-9 activity and impairing CTB-mediated invasion remodeling, which can restrict fetal growth (67). HCMV can infect a broad range of cells and tissue, and infection in CTBs can affect fetal development. Placental pericytes (which are pluripotent cellular components of the capillaries and post-capillary venules) support HCMV replication and may contribute to viral dissemination, triggering inflammation and dysregulation of placental angiogenesis. The retina and brain have the highest density of vascular pericytes in the body (68).

We refer the reader to a recent review by Njue et al. who proposed mechanisms of HCMV-induced placental dysfunction that may have a role in associated still- and preterm birth, and IUGR from in vitro studies in which laboratory-adapted and low-passage strains of HCMV and various human placental models were used. Potential mechanisms identified included impairment of trophoblast progenitor stem cell differentiation and function, impairment of EVT invasiveness, dysregulation of Wnt signaling pathways in CTBs, TNF-α- mediated apoptosis of trophoblasts, HCMV-induced cytokine changes in the placenta, inhibition of indoleamine 2,3-dioxygenase activity, and downregulation of trophoblast class I MHC molecules (69). These in vitro data may provide useful insights into HCMV pathogenesis leading to adverse birth outcomes, but the findings need to be tempered by findings from ex vivo studies with corroboration in suitable animal models, when available.

## ZIKA VIRUS

### Clinical Presentations of Zika Virus Infection

While HIV-1 and HCMV transmission occur through body fluids, which includes saliva for HCMV, blood, and seminal/vaginal secretions, Zika virus (ZIKV) is an arthropod-born virus (arbovirus) transmitted by Aedes aegypti and Aedes albopictus mosquitoes, which are also vectors for dengue and chikungunya viruses. Additionally, ZIKV can be transmitted sexually and vertically (45, 70). ZIKV was first isolated from Rhesus monkeys in 1947. The first patient with ZIKV infection was clinically described in 1954 in Nigeria (70). Serosurvey results from the Yap Island outbreak in 2007 showed that <20% of infected individuals had symptoms traceable to ZIKV. In general, ZIKV infection causes mild symptoms, which persist less than a week. These include fever, maculopapular rash, arthritis, joint pain, conjunctivitis, vomiting, and headaches (71). Rarely, ZIKV infection can manifest with neurologic complications such as Guillain-Barré syndrome.

ZIKV passes through the placental barrier and can replicate in HCs leading to viral dissemination in the fetus with signs of congenital Zika syndrome (CZS), especially following maternal infection in the 1st trimester. Clinical manifestations of CZS include fetal brain disruption sequence leading to microcephaly, subcortical calcifications, hydrocephalus, neuronal migration disorders and brainstem dysfunction. Reported CZS ocular abnormalities include macular scarring and focal retinal pigmentary mottling. Congenital Zika syndrome is also associated with contractures including arthrogryposis.

### Virology and Tropism

Zika virus (ZIKV) belongs to the genus Flavivirus, related to many clinically significant viruses, including dengue, yellow fever, Japanese encephalitis, chikungunya, and West Nile virus. ZIKV is an enveloped virus with single-stranded positive-sense RNA enclosed by an icosahedral nucleocapsid (72). ZIKV encodes three structural proteins; capsid, pre-membrane, and envelope, and seven non-structural proteins; NS1, NS2A, NS2B, NS3, NS4A, NS4B, and NS5.

ZIKV has a broad range of cell tropism and ability to infect tissue in the central nervous system, blood, retinal, and genital and reproductive tract including the placenta (73–77). Specific cells and tissue include HCs, trophoblasts (including EVTs), endothelial cells, neuronal and trophoblast progenitor cells, fibroblasts, matured neuronal cells, astrocytes, and ocular tissue (73). Cell entry is via receptor-mediated endocytosis, and AXL (receptor tyrosine kinase) and T cell immunoglobulin mucin domain 1 (TIM1) are potential receptors that ZIKV utilizes. Following access to fetal blood, AXL-expressing human umbilical vein endothelial cells may facilitate dissemination to the fetal side of the placenta (72). Vertical transmission of ZIKV may occur by: 1) infection of maternal microvasculature and EVTs, 2) infection in maternal immune cells with viral passage through the placenta by passive diffusion or transcytosis, 3) infection-induced damage in the villous tree in the STB layer, and 4) transvaginal ascending infection (78).

### The Role of Hofbauer Cells in Zika Infection

Despite the dramatic presentation of CZS, we have limited knowledge on the pathogenesis of ZIKV at the MFI, reflecting the relatively short time frame of reporting this condition from the initial outbreak in the Americas in 2015. Our group initially showed that ZIKV can infect HCs. Infected HCs may disseminate ZIKV to the fetus. ZIKV replication coincides with induction of type I IFNs, pro-inflammatory cytokines, and antiviral gene expression, but with minimal cell death. These results suggest a “Trojan Horse” mechanism for intrauterine transmission in which ZIKV gains access to the fetal compartment by directly infecting and replicating within HCs, without direct cytotoxicity. Viral replication within HCs was mediated through FcR, TLR4 and DC-SIGN receptors. Infection appears to be augmented in HCs by IgG from prior flavivirus exposure through antibody dependent enhancement (ADE) (79). Multiple studies suggest HCs have a pivotal role in MTCT of ZIKV and are preferentially infected compared to CTBs (80–82). A shortcoming with these in vitro studies is that infection with ZIKV occurred on term HCs. Given our previous work showing how HCs change their phenotype during gestation (10) and that CZS is most often associated with maternal infection in the first trimester, similar studies need to be undertaken in early trimester immune placental cells.

### Placental Innate Immunity and ZIKV Infection

**FIGURE 6 |:**
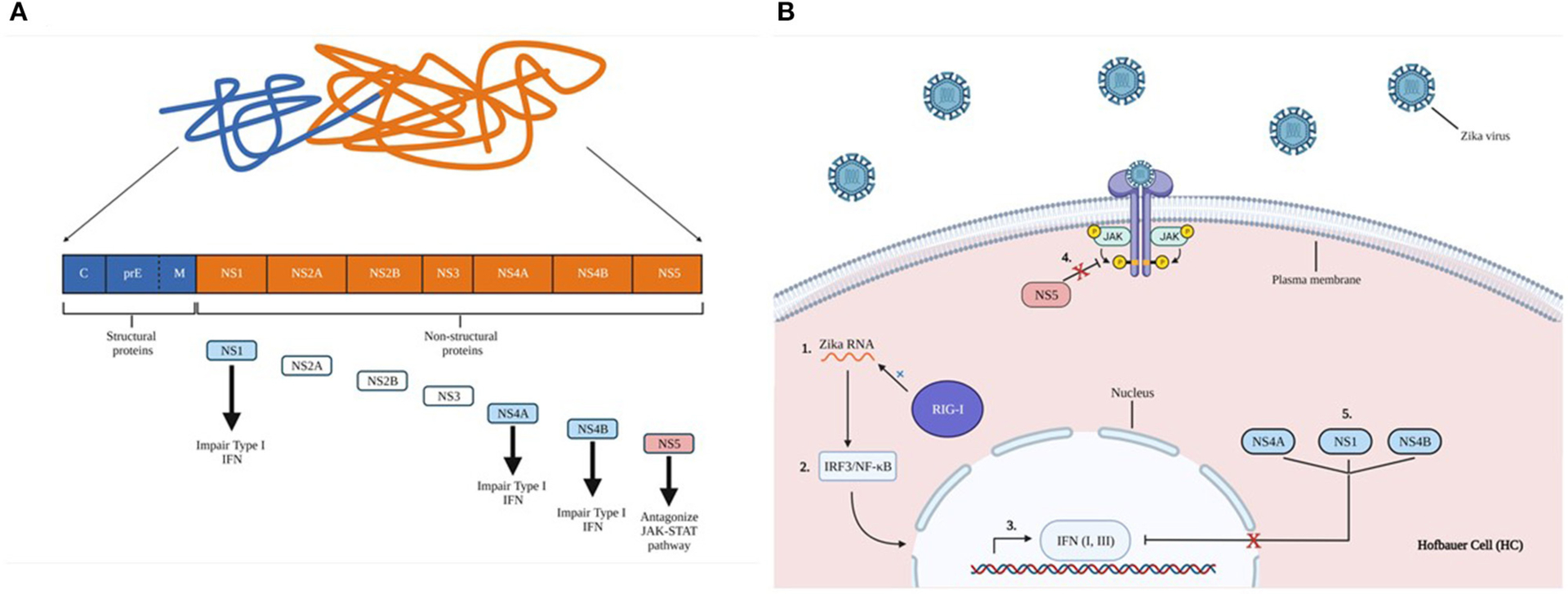
Zika virus non-structural protein impairs innate immune activation. **(A)** Proteolytic cleavage of zika virus polyprotein. Structural proteins form capsids (C), glycoprotein precursor premembrane (PreM), and envelope proteins (E). Structural proteins are indicated in blue, non-structural proteins are shown in orange. A brief description of each essential non-structural protein are indicated by the arrows. **(B)** 1) ZIKV RNA is recognized by the retinoic acid-inducible gene I (RIG-1), 2) leading to subsequent activation of IFN regulatory factors such as NF-kB and IRF3. 3) These activate the production of type I and III IFNs, which are mediated by the JAK-STAT signaling pathway to induce cells into an antiviral state. 4) ZIKV non-structural protein NS5 can antagonize JAK-STAT signaling, evading gene activation, and blocking cellular conversion into an antiviral state. 5) NS1, NS4A, NS4B impair type-I IFN responses.

Innate immune activation against a given virus is associated with viral RNA recognition in the infected cytoplasm by RIG-I, with subsequent activation of IRF3 and NF-kB. IRF3 activation triggers production of type I and III IFNs. This cascade is mediated by the JAK-STAT signaling pathway to activate IFN-stimulating genes (ISGs) to further induce nearby cells to an antiviral state, specifically targeting viral replication. However, ZIKV may be able to evade the innate immune response via the non-structural protein NS5, which can act as an antagonist for the JAK-STAT pathway (83, 84). ZIKV NS1, NS4A, NS4B have been shown to impair type-I IFN responses. TANK binding kinase-1 (TBK1)/IRF3 and JAK-STAT signaling may reduce activation of the innate immune response against ZIKV (Figure 6) (85). Although many innate and adaptive immune responses against ZIKV at the MFI are uncharacterized, ZIKV infection can elicit peripheral T, NK, and B cell responses.

## SUMMARY

In this review, we have focused on three clinically significant viral pathogens during pregnancy. Characterizing placental immunity is exceptionally challenging given that this unique organ constructed from both maternal and fetal tissue, exhibits immunological responses that are markedly different from those characterized in peripheral blood. Against this backdrop, viruses themselves exhibit uniquely specific cell and tissue tropism and evolutionary adaptation, with limited opportunity to study affected cells over gestation in vivo. At the same time, animal models infrequently recapitulate all aspects of human disease.

Immunoquiescence is key to protecting the haploidentical fetus from maternal immunity during gestation. This unique placental milieu is balanced against maintenance of an adequate level of immune protection against invasive perinatal or TORCHESZ infections (T: toxoplasma, O: others such as parvovirus B19, R: rubella, C: CMV, H: herpes simplex virus, hepatitis B, HIV-1, E: enterovirus; S: syphilis, Z: ZIKV). Maternal viral infection during pregnancy can be self-limiting. However, if invasive HCMV infection develops during embryogenesis, teratogenic effects to the developing fetus can occur before diagnosis is established and effective antivirals administered. Furthermore, administering newer antivirals in the first and early second trimester is associated with a potential risk of antiviral-associated teratogenicity. Some antivirals, such as the protease inhibitors used for treatment of HIV-1 infection, should generally be avoided during pregnancy because of an associated risk of preterm delivery (34, 42, 43).

The modest amount of information of human host-placental cell-viral interaction that has been published are mainly from *in vitro* experiments and point to a combination of immunoquiescent factors such as a predominance of regulatory cytokines (IL-10 and TGF-β) at the MFI that are important in not only supporting tolerance to the haploidentical fetus but also limiting inflammation and viral replication during maternal infection. Despite expression of CD4, CCR5, CXCR4, and DC-SIGN, HCs kinetically exhibit slow transcription and replication of HIV-1 compared to MDMs. We postulate that these cells have evolved to capture and contain HIV-1 and other retroviruses over millennia and can limit viral replication through intracellular neutralization and endogenous production and secretion of IL-10 and TGF-β, as shown in Figures 2A,B. The properties of HCs evolve during gestation expressing higher levels of CCR5 and exhibiting an activated phenotype early in pregnancy but being less susceptible to HIV-1 infection compared to term HCs. Perhaps this property reflects increased secretion of anti-inflammatory cytokines, chemokines, and a more robust antiviral immune response in the former. In contrast, term HCs were more susceptible to HIV-1 replication, associated with dampening of IFN-induced STAT1 and STAT2 activation. Viral recognition and antiviral immunity in early gestation HCs may prevent intrauterine HIV-1 infection, whereas diminished antiviral responses at term may facilitate MTCT of HIV-1 and explain why most intrauterine MTCT occurs in the third trimester.

There is emerging clinical data associating HCMV with MTCT of HIV-1 (35, 36, 86), with one study suggesting fetal HCMV infection may predispose infants to intrauterine HIV-1 infection (37). Our supportive studies show that HCMV coinfection enhances susceptibility and viral replication of HIV-1 in HCs. Consistent with enhanced viral susceptibility, HCMV exposure upregulates CCR5 and CD80 expression. HCMV significantly induces type I IFNs, proinflammatory cytokines, and antiviral gene expression. HCMV also reduced type I IFN-mediated phosphorylation of STAT2, resulting in diminished antiviral responses.

CTBs are important sites for placental HCMV infection and most likely transmit virus to the fetus. Cell entry may involve interaction between viral pentameric complex gH/gL/pUL128-pUL131A and cellular platelet-derived growth factor receptor-α (PDGFRα) expressed on placental trophoblasts. EVTs appear to escape CD8+ T cell recognition through a lack of expression of MHC-I receptors. However, HCs appear to be involved in light of HCMV infection of these macrophages, although their specific role in pathogenesis at the MFI during maternal primary infection or reactivation requires further characterization. First trimester dNK cells were able to clear HCMV-infected cells with activating dNK cell receptors including KIR2DS1, KIR2DS2, KIR2DS5 and KIR3DS1 exhibiting antiviral activity and cytotoxicity. However, term dNK cells exhibited reduced efficacy when responding to HCMV-infected cells.

The dramatic appearance of a ZIKV pandemic in the Americas in 2015 raises as many questions as to why and how cases so precipitously decreased in a relatively short space of time. Perhaps this broad and difficult question is beyond the scope of this review. What we learned in terms of the key role of HCs is their ability to sequester ZIKV following infection with minimal cell death, and how ZIKV is able to evade the innate immune response. These observations speak to evolutionary adaptation of ZIKV with immune cells at the MFI. There are glaring omissions in this review on topics that merit further investigation. Because ZIKV can infect many different cell types, it is challenging to characterize the mechanisms by which this virus gains access to the fetus. Further research is needed to identify a putative route of vertical transmission. Vaccination may be a future consideration, however, since ZIKV is closely related to other Flavivirus families, vaccine-induced cross-reactive antibody and antibody-dependent enhancement may cause unexpected and unwanted outcomes.

The importance of studying mechanisms of pathogenesis of viral infections at the MFI in detail is to enable targeted therapeutic interventions including vaccines to mitigate the adverse outcomes of these infections during pregnancy. Characterizing the complexity of interactions at the MFI across gestation with modulation by viral pathogens can also promote improved understanding of the long-term effects of maternal infection during pregnancy on neonatal and childhood immunity.

As a pediatric infectious disease specialist (RC) from the 1990’s, one of the most rewarding outcomes to personally witness has been our ability to reduce MTCT of HIV-1 to just 1–2%. This contrasts with the large number of infants that were becoming HIV-1-infected prior to the landmark Pediatric AIDS Clinical Trial Group (PACTG) 076 study (26). It is therefore a time of great consternation to learn of the health disparities between HEU and HIV-1-unexposed uninfected children, and the more severe clinical outcomes in the former. Identifying modifiable biological mechanisms at the MFI that contribute to these disparities remains a global health priority.
